# Mortality effects of average education: a multilevel study of small neighbourhoods in rural and urban areas in Norway

**DOI:** 10.1186/1475-9276-8-41

**Published:** 2009-12-09

**Authors:** Øystein Kravdal

**Affiliations:** 1 Norwegian Institute of Public Health, PO Box 4404 Nydalen, 0403 Oslo, Norway; 2 Department of Economics, University of Oslo, PO Box 1095 Blindern, 0317 Oslo, Norway

## Abstract

**Background:**

The intention was to find out whether there was an association between the socio-economic resources in a small neighbourhood ("basic statistical unit"; BSU) and individual mortality, net of individual resources, and whether this association differed between municipalities including a quite large city and others. The possibility of a rural-urban difference in the health effect of community resources has not been checked earlier.

**Methods:**

Discrete-time hazard models for mortality at age 60-89 were estimated for 1990-1992 and 2000-2002, using register data that cover the entire Norwegian population. For each person, the educational level and the municipality and BSU of residence in 1990 and 2000 were known. Average education was computed by aggregating over the individual data. In total, there were about 200000 deaths in more than 13000 BSUs during 5 million person-years of observation.

**Results:**

There was a significant relationship between average education in the BSU and individual mortality, but only in the medium-sized and largest municipalities. The sharpest relationship was seen in the latter, where for example OR per year of education was 0.908 (95% CI 0.887-0.929) in the 1990-92 period. The findings were robust to various alternative specifications.

**Conclusion:**

These results from a large data set are consistent with the idea that neighbourhood socio-economic resources may affect individual mortality, but suggest that distinctions according to population size or density be made in future research and that one should be careful, if focusing on cities, to generalize beyond that setting. With these data, one can only speculate about the reasons for the rural-urban difference. A stronger higher-level spatial segregation in urban areas may be one explanation.

## Background

It is well known that a person's health or mortality is related to her or his own socio-economic resources [1]. In addition, many recent multilevel studies from a variety of countries [2,3], including the Nordic ones [4-7], have suggested that, among persons who themselves have the same level of resources, those who live in a community that is relatively advantaged socio-economically have lower mortality and better health than others. Such effects have not shown up in all investigations, though [8-11]. There is little knowledge about the pathways linking individual mortality and community resources [12], but it seems at least reasonable to assume that they involve effects of social interaction with other persons as well as more general effects of the community environment, which others have contributed to build up (see elaboration below). In addition, there may of course be common factors behind individual mortality and community resources.

The level of aggregation has varied greatly across the multilevel studies, from US states [13] or clusters of municipalities [14-16], to areas such as census tracts or city boroughs [4,6,17,18] or even census block groups or parishes with only about 1000-2000 persons [5,19-22]. There are also a few studies that have been based on British census enumeration districts or other small areas with as little as 500 inhabitants [9,23,24], and some have considered more than one level of aggregation [25,26]. It is indeed not obvious what the most appropriate level would be. Much social interaction with others takes place within a neighbourhood, while other relevant mechanisms operate at a higher level (see below). Some results suggest that the community effects are sharpest at a relatively low level [27], but the empirical picture is far from clear.

In some earlier studies, the possibility of cross-level interactions has been addressed. For example, it has been checked whether the association between individual health or mortality and community resources depends on the person's own age [4,16], sex [18,28], socio-economic resources [24,26,29,30] or marital status [31]. In addition, interactions with community-level social cohesion have been analysed [23], and it was reported in a Dutch study, without speculating about explanations, that effects varied across the largest cities [32]. Also variations across countries have been assessed [33].

Several analyses have been confined to single cities, for example Copenhagen [6], Stockholm [34], Helsinki [4,17], Amsterdam [27], London [17], or Chicago [35], or groups of cities or urban census tracts [29,30]. This may reflect an idea that community effects are likely to be sharpest in urban areas, but such a pattern is not documented in the literature. In fact, Sampson [36] pointed out in a review that it is important also to study neighbourhood effects in suburbs and rural areas.

The objective of the present study was to find out whether the effect of community socio-economic resources on individual mortality (or, rather, the relationship between the two, since one cannot expect to be able to control perfectly for selection) indeed is stronger in urban than in rural areas, using nation-wide Norwegian register data where the so-called "basic statistical unit" (BSU) was the level of aggregation. There are more than 13000 such units in the country, having about 350 inhabitants on average. All Norwegian women and men at age 60-89 (currenty, only 7% die at lower ages [37]; see also comment on age restriction below) were followed over two three-year periods, 1990-1992 and 2000-2002 (see motivation for the choice of study periods below). Because few earlier studies have used such a low level of aggregation (in fact none quite as low), and none of them have been based on such a large data set for an entire country, even the overall effects - without regard to the rural-urban differences - should be of interest. Average education was used as the only indicator of community socio-economic resources. It has been included also in earlier multilevel health analyses, alone or in combination with other community variables [5,29,38] or as one of several factors in a socio-economic index [26,30,39]. Education is of course closely correlated with income, and especially at the aggregate level [40], so the estimated effects of education also reflect income effects. More precisely, community education affects mortality partly through community income and partly through various non-economic pathways (see discussion below). In addition, community education is to some extent determined by community income.

## Methods

### Municipalities and basic statistical units (BSUs)

The municipality is the lowest political-administrative unit in the country, except that a few very large municipalities have transferred some authority to boroughs. There were 433 municipalities when the data were compiled (currently 430). Their size differs greatly. Oslo has about half a million inhabitants, and there are 4 other urban municipalities with 100000 - 250000 inhabitants. Among the others, the average population size is 7000, with a variation from 200 to 75000. The largest of them include a city, while the smallest are typically rural.

Each municipality includes a number of BSUs (varying from 2 in the smallest municipality to about 500 in Oslo), which are used in the production of statistics. In total, there were 13277 BSUs in the 1990-92 analysis. The average population size of each BSU is larger in the largest municipalities than in the smaller ones (e.g. 641 persons at age 30-89 in the 10 largest municipalities and 156 in the 100 smallest) and the average education is higher (e.g. one year higher in the 10 largest municipalities than in the 100 smallest).

### Data

The data covered the period up through 2002. They were extracted from the Norwegian population register, which includes everyone who has ever lived in Norway after 1960, and education files from Statistics Norway based on censuses and schools' reporting. For each person, there was information about municipality of residence 1 January every year since 1965, defined not by the official municipality identifiers that could have been used to add more municipality data from other sources, but by internally consistent codes that could be used to construct municipality averages of education and other characteristics by aggregation. The BSUs where the person lived in January 1990 and 2000 were defined by a similar set of confidentiality-preserving codes (i.e. it was possible to calculate average education and other aggregate measures for each BSU, and the researcher knows whether two BSUs are in the same municipality, but not which municipality this is and not whether the two BUSs are adjacent to each other or in different parts of the municipality). Further, there was information about the highest educational level achieved as of October every year from 1980, dates of in- or out-migration, and date of death. The analysis was restricted to 1990-1992 and 2000-2002 and to women and men who lived in Norway 1 January 1990 or 1 January 2000.

The choice of study periods was motivated by practical concerns. When random effects models (see below) are estimated from these Norwegian register data that cover complete cohorts, the standard multilevel software does not allow many years of observation to be included. Further, when using a short observation period such as three years, it would be sufficient to consider only the place of residence at the beginning of the first year, making data construction easier. To strengthen the analysis, while focusing on relatively recent patterns, two three-year periods were considered, one including the three last years covered by the data and one 10 years earlier.

### Estimation of discrete-time hazard models

The procedure described below is for the 1990-1992 analysis. The 2000-2002 analysis was similar. For each person who lived in Norway in the beginning of 1990, a series of one-year observations was created, starting in January 1990 (if born 1901-1930) or in January the year the person turned 60 (if born 1931-1932). Those born 1933 or later did not contribute to the analysis. End of follow-up was at the end of 1992, the end of the year when the person turned 89, or at the time of death or last emigration, whatever came first. One-year observations starting when the person was temporarily abroad were ignored. Within the remaining 2690542 observations, there were 102682 deaths.

Mathematically, the model was

where p_irst _is the probability that person i who lived in BSU r in municipality s at the beginning of year 1990 and still was alive in the country at the beginning of year t (1990-1992) dies within year t. X_irst _is a vector of characteristics of the person at the start of t, V_rs _is a vector of characteristics of the BSU (measured in 1990), and W_s _is a vector of characteristics of the municipality (also in 1990). β_1_, β_2 _and β_3 _are corresponding effect vectors. It was not necessary to include a period term.

In accordance with common practice in multilevel modelling [41], random terms at the BSU (τ_rs_) and municipality level (υ_s_) were included to account for unobserved characteristics at those levels. This increases standard errors of effects at those levels, but has minor impact on point estimates. The MLwiN software (version 2.01) was used for the estimation. Odds ratios with confidence intervals were calculated manually from the reported β estimates and their standard errors. Most models were estimated separately for three groups of municipalities, each including about 1/3 of the deaths in the total material: the 339 municipalities with less than 2000 inhabitants at age 60-89 (33213 deaths), the 82 municipalities with 2000-8000 inhabitants at that age (34947 deaths), and the 12 municipalities with more than 8000 inhabitants (34517 deaths). In these groups, there were 6021, 4597 and 2659 BSUs, respectively. The same cut-points were used in the analysis of 2000-2002, when the number of deaths in the three groups were 30798, 32272 and 31221, respectively, and the total exposure time was 2582427 person-years.

It should be noted that the restriction of the analysis to age 60-89 is not critical. In fact, the pattern in the estimates was the same when persons aged 30-59 were considered instead (not shown).

### Education variables

The education variable referred to the highest level attained as of October the year t-1. Five educational levels were defined [42]: i) compulsory (10 years), ii) lower secondary (11-12 years), iii) upper secondary (13 years), iv) some college (14-17), and v) higher education (18 years or more). The distribution over the educational levels is shown in Table 1.

**Table 1:** Summary statistics and effects of individual and aggregate-level variables on individual mortality among women and men aged 60-89 in 1990-1992 (odds ratios with 95% confidence intervals)^a^

	Estimated effects (odds ratios with 95% CI)		Proportion of observations in this category (%)
Individual variables			
			
Age			
60-64^b^	1		21.5
65-69	1.594****	(1.546, 1.644)	22.8
70-74	2.625****	(2.552, 2.702)	21.4
75-79	4.476****	(4.353, 4.604)	16.7
80-84	7.701****	(7.489, 7.918)	11.5
85-89	13.405****	(13.024, 13.797)	6.1
			
Education			
10 years^b^	1		56.2
11-12 years	0.855****	(0.842, 0.868)	28.3
13 years	0.830****	(0.806. 0.853)	6.9
14-17 years	0.718****	(0.695, 0.741)	6.6
18^+ ^years	0.665****	(0.632, 0.700)	2.1
			
Sex			
Men^b^	1		43.9
Women	0.541****	(0.534, 0.548)	56.1
			
Characteristics of the BSU in 1990			Mean (and standard deviation) across observations
			
Log of population size	1.066****	(1.053, 1.080)	5.56 (0.87)
Average age	1.009****	(1.007, 1.012)	54.90 (4.25)
Average education	0.949***	(0.934, 0.964)	11.56 (0.74)
			
Characteristics of the municipality in 1990			
			
Log of population size	1.027****	(1.014, 1.040)	8.61 (1.63)

* p < 0.10; ** p < 0.05; *** p < 0.01; **** p < 0.001 two-sided

^a ^The model also included one random term at the BSU level and one at the municipality level. Their variances were 0.0448 (with standard error 0.0022) and 0.0069 (with standard error 0.0011), respectively

^b ^Reference category

Unless otherwise stated, the number of years of education (set to 10, 11, 13, 15, and 18 years in the 5 categories; using 16 and 19 instead of 15 and 18 gave essentially the same results) was averaged over all women and men of age 30-89 in the BSU. This large age group was chosen because of the small population in several BSUs. In 1990, the average education varied between 10.0 and 16.5 years, with a mean of 11.6 and a standard deviation of 0.74. As a robustness check, some models included the average over the age groups 30-59 or 60-89 instead of 30-89.

### Control variables

An urban environment may stimulate schooling and attract educated people and also increase mortality. Accordingly, some analyses of the relationship between community socio-economic resources and individual mortality have shown that it is very important to control for urbanization [20]. In the present study, the logarithm of the population size of the BSU (at age 30-89) and the logarithm of the population size of the municipality (age 60-89) were included. The former was particularly important, and especially in the models for the smallest municipalities. Similar results were found if population size instead was entered as 10-level categorical variables.

Also the average age in the BSU (negatively associated with average education) was included, which turned out to be quite important. Further, individual age was included as a categorical variable with 5-year groups. A finer categorization gave the same results. Sex was included in most models.

Measures of the distributions of these variables are shown in Table 1.

## Results

In 1990-1992, individual mortality was inversely related to the average education in the BSU, given individual education (Table 1). The relationship was significant only in the medium-sized and largest municipalities, and clearly sharpest in the latter (Panel A, Table 2). The use of a categorical variable showed that the effect in the largest municipalities was quite monotonic (Panel B, Table 2). In these municipalities, a two-standard-deviation increase in average education would reduce mortality by 13 percent (=1-0.908^2·0.74^) if we give the estimates a causal interpretation.

**Table 2:** Effects of average education in the BSU on individual mortality among women and men aged 60-89 in 1990-1992 (odds ratios with 95% confidence intervals)^a^

	Smallest municipalities		Medium-sized municipalities		Largest municipalities	
Panel A:						
Average education in BSU	1.016	(0.980, 1.052)	0.970**	(0.942, 1.000)	0.908****	(0.887, 0.929)
Panel B:						
Average education in BSU						
< 10.9 years	0.993	(0.957, 1.031)	1.006	(0.962, 1.052)	1.122***	(1.037, 1.214)
10.9-11.2 years	0.983	(0.944, 1.022)	0.991	(0.947, 1.037)	0.989	(0.921, 1.062)
11.2-11.4 years ^b^	1		1		1	
11.4-11.7 years	1.025	(0.983, 1.071)	0.993	(0.950, 1.036)	0.989	(0.936, 1.045)
11.7-12.2 years	1.053**	(1.001, 1.109)	1.010	(0.965, 1.057)	0.956**	(0.906, 1.008)
> 12.2 years	0.977	(0.893, 1.069)	0.943**	(0.891, 0.999)	0.870****	(0.825, 0.915)

* p < 0.10; ** p < 0.05; *** p < 0.01; **** p < 0.001 two-sided

^a ^The models also included individual education, age, sex, the logarithm of the population size in the BSU, the average age of the population in the BSU, the logarithm of the population size in the municipality, a random term at the BSU level, and a random term at the municipality level. In panel A, the variances of the random effects at BSU and municipality level were 0.0264 and 0.0113, respectively, in the smallest municipalities, 0.0506 and 0.0045 in the medium-sized municipalities, and 0.0499 and 0.0023 in the largest municipalities.

^b ^Reference category

Excluding observations from BSUs with less than 50 persons at age 30-89 reduced the data sets by 1-5%. The estimates were essentially unchanged (Panel A, Table 3). When average education at age 30-59 was included instead of that at age 30-89 (and average age also was calculated for that interval), the effect in the largest municipalities was more weakly negative, and that in the medium-sized municipalities was no longer significant. Using the average at age 60-89 gave results more similar to those obtained with age 30-89. There were quite small sex differences (Panel B, Table 3).

**Table 3:** Effects of average education in the BSU on individual mortality among women and men aged 60-89 in 1990-1992 (odds ratios with 95% confidence intervals), among various sub-groups and according to various models^a^

	Smallest municipalities		Medium-sized municipalities		Largest municipalities	
Panel A:						
						
As model in Table 2 but:						
Exclude BSUs with less than 50 persons at age 30-89	1.012	(0.975, 1.050)	0.966**	(0.937, 0.996)	0.904****	(0.872, 0.938)
Average education 30-59 instead of 30-89^b^	1.012	(0.984, 1.091)	0.997	(0.971, 1.023)	0.936****	(0.915, 0.957)
Average education 60-89 instead of 30-89^c^	0.989	(0.949, 1.030)	0.966**	(0.937, 0.996)	0.916****	(0.897, 0.936)
						
Panel B:						
Women	1.009	(0.961, 1.060)	0.951**	(0.913, 0.992)	0.903****	(0.875, 0.932)
Men	1.012	(0.968, 1.058)	0.976	(0.942, 1.012)	0.911****	(0.888, 0.936)

* p < 0.10; ** p < 0.05; *** p < 0.01; **** p < 0.001 two-sided

^a ^The models also included individual education, age, sex (only Panel A), the logarithm of the population size in the BSU, the average age of the population in the BSU, the logarithm of the population size in the municipality, a random term at the BSU level, and a random term at the municipality level.

^b ^Average age also calculated within age 30-59

^c ^Average age also calculated within age 60-89

Finally, the analysis was repeated for 2000-2002. The results were similar, except that a positive effect appeared for women in the smallest municipalities (Table 4).

**Table 4:** Effects of average education in the BSU on individual mortality among women and men aged 60-89 in 2000-2002 (odds ratios with 95% confidence intervals), among various sub-groups and according to various models^a^

	Smallest municipalities		Medium-sized municipalities		Largest municipalities	
Both sexes	1.041*	(1.000, 1.084)	0.964**	(0.934, 0.996)	0.894****	(0.872, 0.916)
Women	1.087***	(1.028, 1.149)	0.961*	(0.920, 1.004)	0.901****	(0.872, 0.931)
Men	0.995	(0.947, 1.045)	0.961**	(0.926, 0.997)	0.889****	(0.864, 0.914)
						
Both sexes, but						
Average education 30-59 instead of 30-89^b^	1.005	(0.971, 1.039)	0.983	(0.953, 1.013)	0.919****	(0.897, 0.941)
Average education 60-89 instead of 30-89^c^	1.037*	(0.996, 1.080)	0.990	(0.961, 1.019)	0.909****	(0.890, 0.929)

* p < 0.10; ** p < 0.05; *** p < 0.01; **** p < 0.001 two-sided

^a ^The models also included individual education, age, sex (in some of the models), the logarithm of the population size in the BSU, the average age of the population in the BSU, the logarithm of the population size in the municipality, a random term at the BSU level, and a random term at the municipality level.

^b ^Average age also calculated within age 30-59

^c ^Average age also calculated within age 60-89

## Discussion

### General mechanisms

To facilitate the discussion of the rural-urban differences, some general causal mechanisms are first briefly reviewed, with special attention to the level at which they may play out in the Norwegian setting.

One reason why one might expect an effect of community education is that better-educated people may have learned about health at school; they may have become more conscious about the ability to influence their health; and their skills and credentials may have given them a higher income, in turn facilitating health-promoting activities [43]. This knowledge, attitude and behaviour may be passed on to others through social interaction [44]. It is not easy to define an appropriate level of aggregation for this mechanism. One typically interacts directly with a subgroup of people in the neighbourhood, who in turn interact with others. In addition, there is direct interaction with persons outside the immediate neighbourhood, and one observes other people's lifestyle in a more anonymous way through wide-covering media.

A second type of pathway that in principle may be relevant is that the higher average income resulting from higher education among people in the neighbourhood may contribute to a more pleasant physical environment, which may encourage outdoor physical activities and perhaps produce a general feeling of well-being. (In some countries, low crime rates in the richest neighbourhoods may add to the advantage.) Again, a broader area is also of relevance. One may benefit from the qualities of other areas not too far way, and if there are rich people in other parts of the municipality there will be higher incomes from taxation to spend on upgrading of all neighbourhoods in the municipality.

A third possible mechanism, operating in particular at the municipality level or even higher, is that the education of other people might affect the quality of the health services. As a background for this argument, some basic facts about the Norwegian health care system are necessary: The municipalities are responsible for primary health care, including for example health centres with general practitioners and nursing homes [45]. These services are financed by the municipalities' tax revenues, various types of transfers from the national government (grants to the municipality, partly to compensate for low levels of local revenues, and reimbursements for each patient from the national social security system), and relatively small payments from the patients. In addition, many general practitioners operate on a private basis, but with much support from the municipality and the national government. Specialist health services are to an even larger extent public. During the period under study, the vast majority of the hospitals - each of which had responsibility for a certain group of municipalities - were owned by and financed by the government or the counties (the incomes of the latter stemming from taxation of their citizens and government grants). The additional private hospitals or individual specialists receive support from the government. Returning to the importance of socio-economic resources, it may possibly be easier to recruit qualified personnel to the various private and public health services in (or with special responsibility for) the municipality under consideration when many persons in that municipality or nearby are well educated. One might also expect that higher tax incomes in the municipality would contribute to a higher density of public health centres or nursing homes, or that higher purchasing power of the inhabitants might fuel the establishing of private health services, which also the socially less advantaged could benefit from. Unfortunately, there is little knowledge about these potential effects. In one study, a positive association between the economic resources available to the municipality government and the density of primary physicians was suggested, though the mean income level appeared to be unimportant [46].

Fourth, a high level of education within an area that may be considered a local labour market may, of course, not only increase the income of "others", but also increase the chance that the person under consideration has a well-paid job and thus a high retirement pension later. A high income may in turn reduce mortality [47] because of its implications for the person's health behaviour or (though less relevant in a society with a public health care system) his or her access to good health care.

A fifth possible mechanism, through probably less important, is the following: When other people have better health because of better education, and therefore present less competing demand for health services, the individual under consideration may receive better help. Given the organization of the health care system, the municipality level is especially relevant, but the competing demand in a broader area may also have some importance, and for nursing homes in the largest cities, the catchment area is often one or more boroughs.

Sixth, there may be an offsetting mechanism contributing to an *adverse *effect of community education: If we compare among persons with the same level of education who live in different areas, those who live in neighbourhoods with a high average education have a lower education *relative *to others in the neighbourhood than do those who live in neighbourhoods were people are not so well educated. It has been argued that a low relative *income *may produce a psychosocial stress that increases mortality [15,48-51], and perhaps a low relative *education *has a similar impact, although it is typically less visible. Just as for the learning argument, a relevant level of aggregation is difficult to define.

### Possible reasons for differences between small and large municipalities?

The argument about pleasant physical environments is probably not very important in Norway, where even the poorest neighbourhoods do not look too bad, and green and perhaps quite unspoiled areas are never far away. It is of particularly little importance in the more rural areas. Below, it is discussed whether also the other causal pathways suggested above might be less powerful in the less populated (and more rural) municipalities.

Let us first consider the mechanisms involving social interaction or comparison with others, which are particularly likely to operate at a low level of aggregation. In the smallest municipalities, the BSUs tend to include fewer persons and cover a larger area than in the larger municipalities. Should we expect that the influence from other people in the same BSU, through learning, imitation or comparison, is weaker when there are fewer of these people and the distance to them is larger? That is far from obvious. For example, while it may be easier to meet people when distances are short, high population density may also strengthen the need for privacy. Empirical studies have provided mixed conclusions. Some have suggested that low population density reduces the amount of social interaction [52], while others have pointed in the opposite direction [53].

The other mechanisms probably operate largely at a higher level, i.e. also the socio-economic characteristics of neighbouring BSUs may affect mortality [54-56] through these pathways. Unfortunately, there is no information about neighbouring BSUs in the data (see comment on BSU identifiers above), but it seems reasonable to base the discussion on an assumption that there is some clustering, in the sense that low-education BSUs in a municipality are more likely than the high-education BSUs to have low-education BSUs as neighbours.

Let us first assume that the degree of clustering is the same throughout the country. Certainly, the generally higher educational level in the large municipalities means that low-education BSUs in small municipalities are more likely to have low-education BSUs as neighbours than are the low-education BSUs in large municipalities. However, that is the case also for high-education BSUs. The assumption about homogenous clustering means that the difference between low- and high-education BSUs in the proportion low-education BSUs among their neighbours is the same for small and large municipalities. In such a situation, one possible reason for the pattern in the estimates may be the following: In the largest municipalities, the neighbouring BSUs within the area under consideration may have a large enough population to function as a local labour market or a catchment area for health institutions, making the arguments above about well-paid jobs and high-quality health care particularly relevant. In contrast, these factors may be influenced by BSUs farther away, which may be more different, in the smaller municipalities. (The social interaction mechanisms also involve this higher level of aggregation [57], but as mentioned earlier, and with relevance also at a higher level, the interaction with population density is not obvious.)

The other possibility is that the degree of clustering actually does differ between small and large municipalities, so that the health of those who live in a low-education BSU in a large municipality is more negatively influenced by characteristics in the wider community than is the case for those living in high-education BSUs, while there is less difference between the neighbours of low- and high-education BSUs in rural areas. That would accord with the sharper relationship between individual mortality and BSU average education observed in the largest than in the smallest small municipalities. Unfortunately, the empirical underpinning for such an idea is weak. There appear to be some differences between the largest Norwegian cities in the degree of spatial segregation above the BSU level [58], but the rural-urban differences have not been checked (and it could not be done with the data available here). Recent American studies based on measures of dissimilarity at different levels of aggregation have not documented any such relationship with population size either [59,60].

### Confounders

In addition to the causal effects discussed so far, the estimates may reflect various selection mechanisms. One is that certain characteristics of the BSU or a larger area that increase people's chances of taking much education or that attract people with high education (e.g. physical environment, economic resources, or cultural values) also may affect mortality. In this study it is only controlled for the population size of the BSU and the municipality and the average age. In principle, the rural-urban differences in the estimates may reflect that there are other additional determinants of education in small than in large municipalities. For example, low-education BSUs in large cities may to a larger extent than low-education BSUs elsewhere be located near major traffic routes, which may increase mortality.

Further, the estimates may be partly a result of *individual *unobserved characteristics because of selective migration. More precisely, people who live in a BSU where the average education is high may be different (beyond what we can measure with the available variables) from those living in other BSUs, and not as a *result *of the high average education, which would simply be a causal pathway, but because some characteristics may increase the chance of moving to or remaining in a place with many better-educated. These characteristics may also affect mortality. For example, one might speculate whether high-class areas in large cities are particularly popular, perhaps because their advantages for some reason are more conspicuous, and therefore attract a special type of "successful" people who would have low mortality anyway.

## Conclusion

Using a low level of aggregation compared to most other studies and a data set with a large number of such units and many observations, this study has supported the idea of an association between community socio-economic resources and individual health and mortality, though there is a far step from this to assuming a causal effect. The relationship is restricted to the largest municipalities. One reason for this may be that also neighbouring BSUs are influential, and that there is a stronger spatial segregation above the BSU level in the urban areas. Put differently, the use of a somewhat higher level of aggregation might have given less pronounced rural-urban differences, and perhaps generally stronger effects. However, there are also other possible reasons for the observed pattern. Further exploration of rural-urban differences, based on data with measurements at several levels, might be worthwhile. If also other studies reveal such differences, it would suggest that distinctions according to population size or density be made in future research and that one should be careful, if focusing on cities, to generalize beyond that setting.

## Competing interests

The author declares that he has no competing interests.
